# Use of Zinc Oxide Nanoparticles as Anticoccidial Agents in Broiler Chickens along with Its Impact on Growth Performance, Antioxidant Status, and Hematobiochemical Profile

**DOI:** 10.3390/life12010074

**Published:** 2022-01-05

**Authors:** Zeynab Khamis El-Maddawy, Abd El-salam Fawzy El-sawy, Neveen Ragab Ashoura, Salama Mostafa Aboelenin, Mohamed Mohamed Soliman, Hany Fawzy Ellakany, Ahmed Ragab Elbestawy, Nahed Ahmed El-Shall

**Affiliations:** 1 Department of Veterinary Pharmacology, Faculty of Veterinary Medicine, Alexandria University, Edfina 22758, Egypt; zeynabelmaddawy@alexu.edu.eg (Z.K.E.-M.); abdelsalam.alsawy@alexu.edu.eg (A.E.-s.F.E.-s.); neven.vet8@alexu.edu.eg (N.R.A.); 2 Biology Department, Turabah University College, Taif University, Taif 21995, Saudi Arabia; s.aboelenin@tu.edu.sa; 3 Clinical Laboratory Sciences Department, Turabah University College, Taif University, Taif 21995, Saudi Arabia; mmsoliman@tu.edu.sa; 4 Department of Poultry and Fish Diseases, Faculty of Veterinary Medicine, Damanhour University, Damanhour 22511, Egypt; ellakany_hany@hotmail.com (H.F.E.); ahmed.elbestawy@vetmed.dmu.edu.eg (A.R.E.); 5 Department of Poultry and Fish Diseases, Faculty of Veterinary Medicine, Alexandria University, Edfina 22758, Egypt

**Keywords:** anticoccidial, zinc oxide, broiler chickens, *Eimeria tenella*, nano particle, carotenoids, nanotechnology, diclazuril, performance, coccidiosis

## Abstract

The impact of zinc oxide nanoparticles (ZnO-NPs) on the pathogenesis of coccidiosis in broiler chickens was tested. A total of 160 1-day-old broiler chicks (Ross 308) were randomly allocated into 4 groups (n = 40). Group 1: unchallenged, unmedicated; Group 2: challenged, unmedicated; Group 3: challenged, supplemented with diclazuril (1 ppm); Group 4: challenged, supplemented with ZnO-NPs (20 ppm). Mixed Eimeria species (*E. maxima*, *E. acervulina*, *E. mivati*, and *E. tenella*) of a commercial coccidial vaccine (FORTEGRA^®^) were used to perform the coccidial challenge by 15× of its vaccinal dose on the 14th day of age. Diclazuril and ZnO-NPs supplementation in Group 3 and 4, respectively, reduced the mortality rate due to coccidial challenge to 5.8% compared to 11.9% in Group 2. The growth performance was improved by ZnO-NPs in coccidiosis-infected group (*p* ≤ 0.05) compared to Group 2 and was comparable to that of Group 3 (*p* ≥ 0.05). The average oocyst count was lower in Groups 3 and 4 (7.8 × 10^3^ and 14.3 × 10^3^, respectively) than in Group 2 (67 × 10^3^ oocysts). Group 3 had a decreased gross lesion score in duodenum and caecum (*p* ≤ 0.05) as well as jujenum and ileum (*p* ≥ 0.05) compared to Group 2; while the average lesion scores of all intestinal parts in Group 4 were significantly decreased (*p* ≤ 0.05). However, diclazuril was superior to ZnO-NPs in reducing caecal lesion score (*p* ≤ 0.05). Plasma carotenoids levels were increased by diclazuril (*p* ≥ 0.05) and ZnO-NPs (*p* ≤ 0.05) supplementation compared to Group 2. Oxidative stress appeared on the fourth week post-challenge (pc) in Group 2 (*p* ≤ 0.05) compared to Group 1, while the dietary supplementation with either diclazuril or ZnO-NPs numerically decreased Malondialdhyde (*p* ≥ 0.05) and statistically increased antioxidant activity (*p* ≤ 0.05). Both medications significantly improved the PCV%, Hb% and RBCs count on the 6th-day and 4th-week pc (*p* ≤ 0.05) compared to Group 2, though this improvement was higher significantly in Group 4 than Group 3 on the 6th day pc (*p* ≤ 0.05). Neither coccidial challenge nor medications had an impact on the total WBCs count as well as organ index, except Bursa of fabricious index that significantly improved by ZnO-NPs on the 4th-week pc compared to Group 2. Coccidial challenge reduced total protein and globulin levels and increased the serum alanine aminotransferase, serum cholesterol, and low-density lipoprotein levels (*p* ≤ 0.05) compared to Group 1, while those of both medicated groups (Group 3 and 4) were comparable to Group 1 (*p* ≥ 0.05). In conclusion, ZnO-NPs were found to be as effective as diclazuril against coccidiosis. However, further research is needed to fully comprehend its anticoccidial mechanisms.

## 1. Introduction

Coccidiosis is a protozoan disease in birds that causes intestinal bleeding, weight loss, a decreased feed conversion ratio (FCR), increased susceptibility to co-infections, and death [1]. Coccidiosis is caused by seven Eimeria (E) species, and each species develops inside a particular part of chicken’s digestive tract. *E. acervulina*, *E. praecox* and *E. mitis* inhabit duodenum and jejunum, *E. necatrix* and *E. maxima* inhabit jejunum and ilieum, while *E. tenella* and *E. brunetti* inhabit the cecum and rectum, respectively. *E. mivati* and *E. hagani* are two other Eimeria species inhabit duodenum, but the validity of them as species is under review [2]. The concurrent infection with several Eimeria species during natural outbreaks at the same time commonly occurs. Manifestations caused by coccidiosis may range from asymptomatic enteric infection to subacute mortality [3]. Beside low growth performance induced by coccidiosis, some other concomitant pathophysiological effects also exert including reduced feed and water intake, increased intestinal acidity and passage time, intestinal malabsorption, reduced digesta viscosity and nutrient digestion, villus atrophy, intestinal leakage of plasma proteins [4]. Therefore, nutritional absorption (minerals, vitamins, glucose, amino acids, and pigments particularly carotenes) is impaired when the duodenum, jejunum, or ileum are infected with Eimeria spp. Furthermore, blood constituents may be affected; for example, *E. tenella* decreased packed cell volume (PCV%) and hemoglobin (Hb%), altered WBCs where lymphocytes decreased and heterophils increased [5] as well as increased liver enzymes, particularly aspartate aminotransferase (AST) [6]. Besides, several studies reported that lipid peroxidation has a role in coccidiosis pathogenesis, thus malondialdehyde (MDA) as an outcome of oxidative stress was increased [7,8,9]. Therefore, coccidiosis is a significant burden to broiler chickens, costing between €2.55 and €2.97/m^2^ of the broiler house if not controlled [10]. Since 1948, coccidial infections have been managed with a variety of synthetic chemical anticoccidial drugs, with ionophorous antibiotics introduced in the 1970s. However, coccidial outbreaks still occur due to drug resistance [11]. Unfortunately, no new medications have been approved for use [12], and this has resulted in an increased demand for novel anticoccidial alternatives. More recently, nanotechnology approaches have been introduced into the veterinary field, not only for disease diagnostics, but also the creation of therapeutics and preventatives [13]. Due to their small size and unique physicochemical features, they are useful in biomedical applications because they enable regulated drug release, targeted drug administration, and in vivo immunomodulation. Interestingly, trace elements nanoparticles have shown antibacterial, antiparasitic, and antioxidant effects. Zinc oxide nanoparticles (ZnO-NPs) have garnered a lot of interest due to size, shape, large surface area, high surface activity, high catalytic efficiency, and strong adsorbing capacity [14]. Wahab et al. [15] investigated the antibacterial activity ZnO-NPs spectroscopically and revealed that 5 μg/mL of non-hydrolytic solution of ZnO-NPs inhibited the growth of K. pneumonia, whereas the growth of E. coli, S. aureus, and S. typhimurium were inhibited by 15 μg/mL of ZnO-NPs. Additionally, ZnO-NPs reduced the deleterious effects of multidrug-resistant Staphylococcus aureus-induced footpad dermatitis in broiler chickens and improved their behavior (standing and walking) [16]. Several reports have demonstrated the antioxidant activities of ZnO-NPs [17,18,19,20]. The anticoccidial effect of ZnO-NPs was investigated *E. stiedae* in rabbits and *E. papillate* in mice [21,22], and indicated that these nanoparticles have a protective effect against coccidiosis. Therefore, in this study, the anticoccidial effect of ZnO-NPs (20 ppm) on broiler chickens experimentally infected with mixed Eimeria species was compared to that of diclazuril (chemical anticoccidial drug), in addition to elucidating its antioxidant and growth-promoting effects.

## 2. Materials and Methods

### 2.1. Experimental Design

The experiment was conducted in the Faculty of Veterinary Medicine, Alexandria University according to the standards of Alexandria University’s Committee of Experimental Animals. Using a humane endpoint protocol, diseased birds suffering for 48 h were euthanized using isoflurane >5%, and cervical dislocation was performed. 

An anticoccidial and antimicrobial drug-free diet, containing zinc oxide 75 mg/kg (basal diet), was provided ad libitum throughout the experiment for all experimental groups (Table 1). A total of 160 1-day-old broiler chicks (Ross 308) were weighed and randomly assigned into 4 groups (n = 40). Group 1: unchallenged, unmedicated (basal diet); Group 2: challenged, unmedicated (basal diet); Group 3: challenged, supplemented with diclazuril (1 ppm) + basal diet; and Group 4: challenged, supplemented with ZnO-NPs (20 ppm) + basal diet. 

### 2.2. Drugs and Coccidial Challenge

Diclazuril, an anticoccidial feed additive, was obtained from Pharma-swede (Egypt). Zinc Oxide nanoparticles (ZnO-NPs) were obtained from Nanotech Egypt (Photo-Electronics Co., 6th October, Giza, Egypt), with average size of 30±5nm, 98% purity, and molecular weight of 81.37. Diclazuril 1 ppm [23] and ZnO-NPs 20 ppm [24] were added to the basal diet of Groups 3 and 4, respectively, and were fed from the 1st to the 42nd day of age.

**Figure 1:**
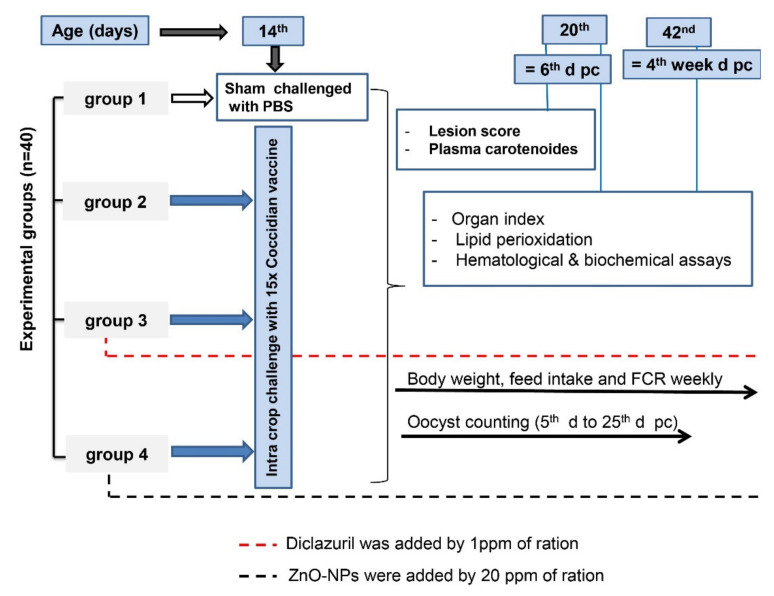
Schematic design of the experiment. PBS: phosphate buffer saline. d pc: day post-challenge. The drugs were supplied to birds in the diet from 1st to 42nd day of age.

The chickens were challenged on the 14th day by intra-crop inoculation using a mixture of Eimeria species obtained from a commercial coccidial live vaccine (FORTEGRA, Intervet Inc. Omaha, NE, USA, U.S. Vet Lic. No. 165A). It was composed of *E. maxima*, *E. acervulina*, *E. mivati*, and *E. tenella*, and each bird received a dose 15 times. Figure 1 shows the experimental design of the study.

### 2.3. Blood Sampling

On the sixth day and fourth week post-challenge, six blood samples were collected per group to obtain plasma for hematological and plasma carotenoid examination. The blood samples were centrifuged at 1500× *g* for 15 min, and sera were collected and stored at −20 °C until biochemical analysis.

### 2.4. Parameters

#### 2.4.1. Plasma Carotenoid Concentration and Antioxidant Activity

Plasma carotenoid concentration was determined spectrophotometrically according to [25]. Serum malondialdehyde (MDA) and antioxidant activity (AOA) were measured according to [26,27].

#### 2.4.2. Parasitological Parameters and Organ-to-Body Weight Index

Six birds/groups were randomly selected and dissected on the sixth day post-challenge to rank the intestinal gross lesions, numerically, caused by coccidiosis in duodenum, jejunum, ilium and cecum according to lesion scoring method of Reid and Johnson [28]. Fresh fecal samples were collected every 48 h from the 5th day to the 25th day post-challenge for oocyst counting using the McMaster slide [29]. The index weights of the liver, kidney, bursa of Fabricius, spleen, heart, and gizzard were calculated as follows: index weight (g\100 g bodyweight) = organ weight/bodyweight × 100.

#### 2.4.3. Mortality Rate and Growth Performance

The chicks were observed daily for deaths. They were individually weighed on the first day, then weekly thereafter. The weight gain of chickens (g), the feed intake, and feed conversion ratio (FCR) were then calculated.

#### 2.4.4. Hematological and Biochemical Analysis

Packed cell volume (PCV%), erythrocytes, and WBC count were measured according to Dacie and Lewwis [30], while hemoglobin concentration (Hb%) was measured according to Winterobe [31]. Serum alanine and aspartate aminotransferase (ALT, AST, respectively), and alkaline phosphatase (ALP) activity were measured according to Reitman and Frankel [32] and Kind and King [33]. Total protein and albumin levels were measured, and serum globulin was calculated by subtracting serum albumin from total protein, according to Doumas et al. [34]. Total cholesterol, high-density lipoprotein (HDL), low-density lipoprotein (LDL)-cholesterol, and triglyceride levels were measured according to [35,36,37,38]. Serum very low-density lipoprotein (VLDL)-cholesterol was estimated using the formula: VLDL-cholesterol = total cholesterol − (HDL-cholesterol + LDL-cholesterol).

### 2.5. Statistical Analysis

It was conducted using ANOVA [39]. The differences were judged significant at *p* ≤ 0.05.

## 3. Results

### 3.1. Plasma Carotenoids and Antioxidant Activity

**Figure 2:**
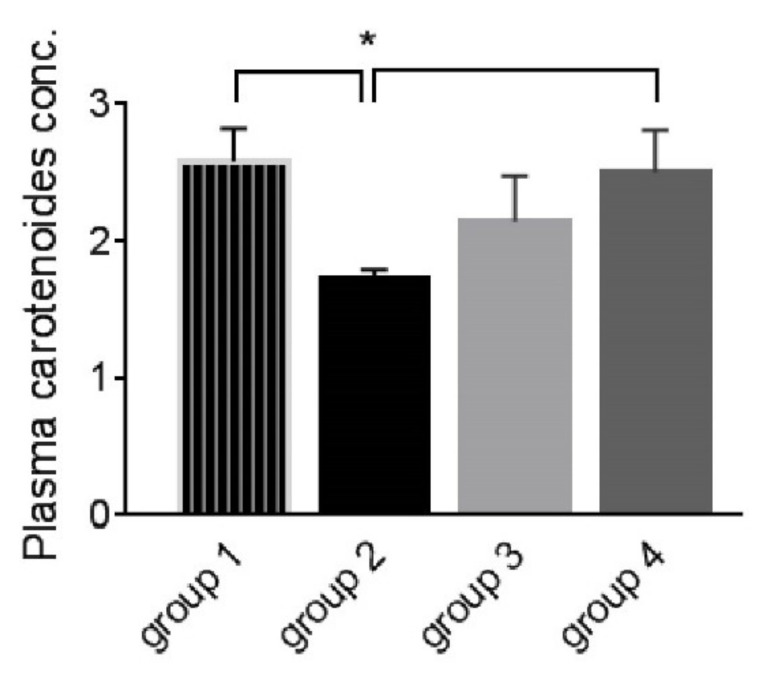
Plasma carotenoid concentration on the sixth day post-coccidial challenge. Asterisk indicates this group (2) is significantly different from groups 1 and 4 (*p* ≤ 0.05). Group 1: unchallenged and unmedicated; Group 2: challenged and unmedicated; Group 3: challenged and supplemented with dicalzuril, 1ppm; and Group 4: challenged and supplemented with ZnO-NPs, 20 ppm.

**Figure 3:**
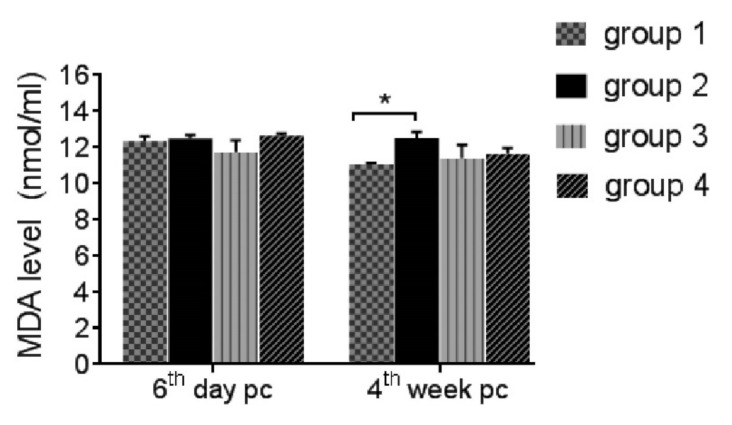
Malondialdhyde (MDA) level on the sixth day and fourth week post-challenge (pc). Asterisk over horizontal line indicates significant difference (*p* ≤ 0.05). Group 1: unchallenged and unmedicated; Group 2: challenged and unmedicated; Group 3: challenged and supplemented with dicalzuril, 1ppm; and Group 4: challenged and supplemented with ZnO-NPs, 20 ppm.

**Figure 4:**
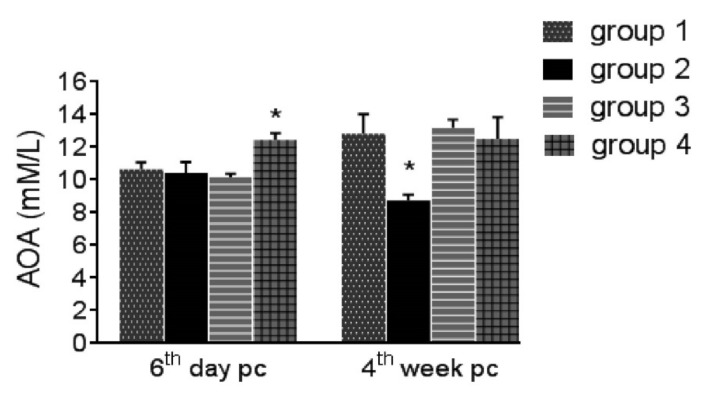
Serum antioxidant activity (AOA) on the 6th day and 4th week post-challenge (pc). Asterisk indicates this group is significantly different from others (*p* ≤ 0.05). Group 1: unchallenged and unmedicated; Group 2: challenged and unmedicated; Group 3: challenged and supplemented with dicalzuril, 1ppm; and Group 4: challenged and supplemented with ZnO-NPs, 20 ppm.

On the 6th day post-challenge, plasma carotenoid levels were decreased in Group 2 than in Group 1 (*p* ≤ 0.05), while they were improved in Groups 3 (*p* ≥ 0.05) and 4 (*p* ≤ 0.05) (Figure 2). No effect was observed on either MAD or AOA on the sixth day post-challenge in all groups except ZnO-NPs had increased AOA significantly during the coccidial infection. On the 4th week post-challenge, coccidial challenge in Group 2 increased MDA levels and decreased AOA significantly comparing to Group 1, while both medications lowered MAD (*p* ≥ 0.05) and increased AOA (*p* ≤ 0.05) (Figure 3 and Figure 4).

### 3.2. Oocyst Shedding, Lesion Score and Organ Index

**Figure 5:**
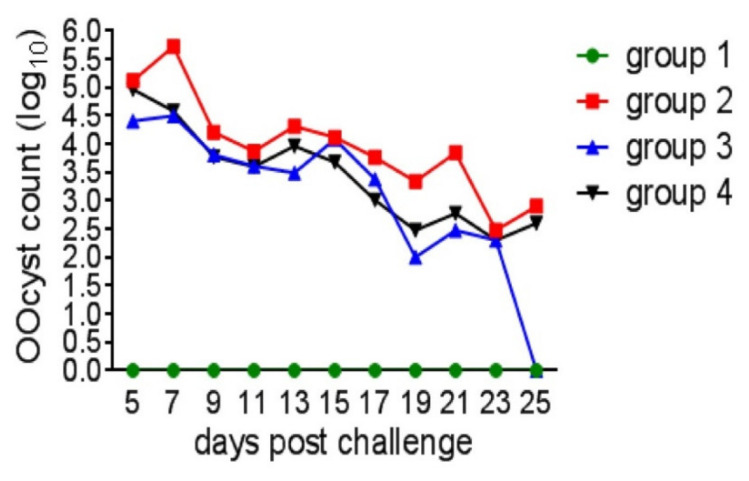
The oocyst counts (log_10_) post-coccidial challenge. Group 1: unchallenged and unmedicated; Group 2: challenged and unmedicated; Group 3: challenged and supplemented with dicalzuril, 1ppm; and Group 4: challenged and supplemented with ZnO-NPs, 20 ppm.

**Figure 6:**
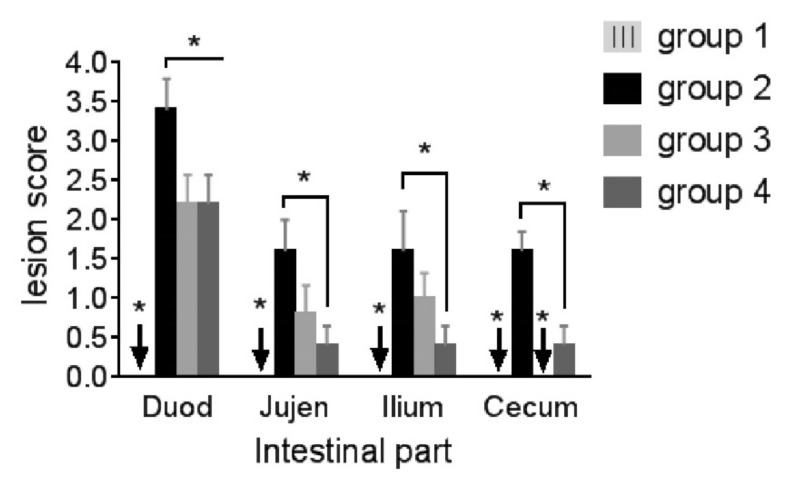
Intestinal lesion scores on the 6th day post-coccidial challenge. Duod: duodenum, Jujen: jejunum. Asterisk over horizontal line indicates significant difference between those groups at this part (*p* ≤ 0.05). Asterisk over arrow indicates significant difference of this group (had lesion score = 0.00) with others (*p* ≤ 0.05). Group 1: unchallenged and unmedicated; Group 2: challenged and unmedicated; Group 3: challenged and supplemented with dicalzuril, 1 ppm; and Group 4: challenged and supplemented with ZnO-NPs, 20 ppm.

The replication potential of coccidial oocysts in Group 2 was observed by counting the fecal oocysts (Figure 5), which had a total average of 3.87 log_10_, along with lesion scores ranging from 1.60 ± 0.25 to 3.40 ± 0.40 (*p* ≤ 0.05) in the four intestinal sections (Figure 6). Oocyst shedding was highest on the 7th day post-challenge in all challenged groups; Groups 3 and 4 had 3.19 and 3.43 log_10_ oocysts, respectively. Additionally, Group 3 had decreased gross lesion score in duodenum and caecum (*p* ≤ 0.05) as well as jujenum and ileum (*p* ≥ 0.05) compared to Group 2; while the average lesion scores of all intestinal parts in Group 4 were significantly decreased (*p* ≤ 0.05). Group 4 had lower score (*p* ≥ 0.05) in the Jejunum and Ilium than Group 3, whereas two ceca in Group 3 had no lesions ((*p* ≤ 0.05) compared to Group 4. Neither intestinal lesions nor oocyst shedding was recorded in Group 1.

All groups had similar index weights for the liver, kidney, bursa of Fabricious, spleen, heart, and gizzard on the sixth day post-challenge. The same was observed on the fourth week post-challenge, except the bursa of Fabricious’ index that was lower in Group 2 (*p* ≥ 0.05) compared to Group 1, whereas it was increased by ZnO-NPs (*p* ≤ 0.05) and diclazuril (*p* ≥ 0.05) supplementation (Table 2).

### 3.3. Mortality % and Growth Performance

**Figure 7:**
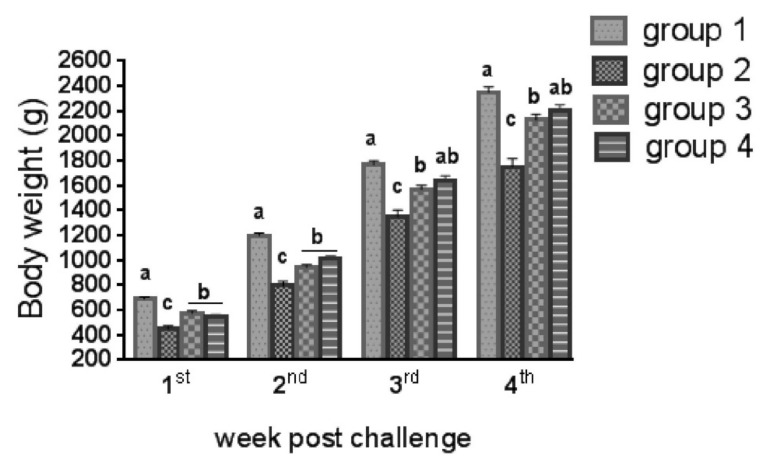
Average body weight (g) at weekly intervals beginning from one week post-challenge. Group 1: unchallenged and unmedicated; Group 2: challenged and unmedicated; Group 3: challenged and supplemented with dicalzuril, 1 ppm; and Group 4: challenged and supplemented with ZnO-NPs, 20 ppm. Different superscript letters indicate significan difference at each time point (*p* ≤ 0.05).

The coccidial challenge resulted in an 11.9% mortality rate in Group 2 compared with 5.8% in the medicated groups (Groups 3 and 4). Moreover, 1 week post-challenge, the body weights began to decrease in all challenged groups (*p* ≤ 0.05) (Figure 7), with Group 2 losing the most weight (54.4% loss). In the following weeks post-challenge, weight loss was 33.9%, 5.4%, and 31%, which were significantly different compared to those of Group 1 (*p* ≤ 0.05). The weekly calculated FCR of Group 2 post-challenge was increased, with a total FCR of 2.15 ± 0.10 that was significantly different compared to that of Group 1 (1.68 ± 0.03) (*p* ≤ 0.05). The total average weight gain of the medicated groups was higher than that of Group 2 (*p* ≤ 0.05) and comparable to that of Group 1 (*p* ≥ 0.05). Furthermore, the same was observed for the FCR (Table 3).

### 3.4. Hematological Picture and Differential Leukocytic Count

The hematological picture (PCV%. Hb%, and RBCs) in Group 2 significantly worsened after the coccidial challenge (Table 4). It was more improved in Group 4 than in Group 3 (*p* ≤ 0.05), and both were comparable to Group 1 (*p* ≥ 0.05). When compared to Group 1, basophils and heterophils in Group 2 were increased (*p* ≤ 0.05), but lymphocytes were decreased significantly (*p* ≤ 0.05) on the sixth day post-challenge (Table 5). Groups 3 and 4 showed an increase in lymphocyte % (*p* ≥ 0.05) as well as a decrease in basophil % (*p* ≥ 0.05) for Group 3; (*p* ≤ 0.05) for Group 4). Except for heterophil %, which diclazuril dramatically lowered, the differential leukocyte counts were similar between the medicated groups. The total WBC counts of all groups did not show significant differences throughout the experiment.

### 3.5. Biochemical Assays

#### 3.5.1. Liver Functions

Neither coccidial challenge nor medication changed the AST, ALT, and total bilirubin levels at both times of observations (6th day and 4th week post-challenge). The ALP level in Group 2 nearly doubled of Group 1 (*p* ≤ 0.05), whereas it was reduced in Groups 3 and 4 (*p* ≤ 0.05) to be comparable to Group 1 on the 6th day and 4th week post-challenge (Table 6).

#### 3.5.2. Serum Proteins

**Figure 8:**
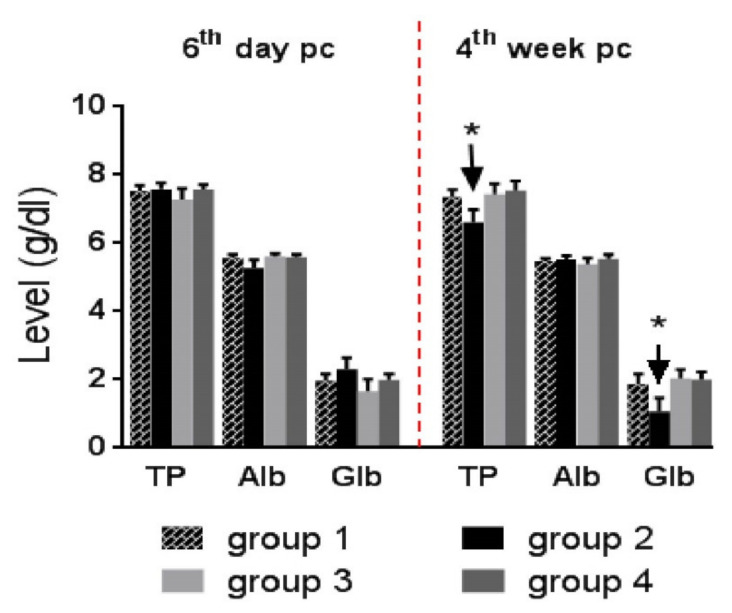
Total protein (TP), Albumin (Alb), and globulin (Glb) levels (g/dL) on the 6th day and 4th week post-coccidial challenge (pc). Asterisk over an arrow indicates significant difference of this group at time points (*p* ≤ 0.05). Group 1: unchallenged and unmedicated; Group 2: challenged and unmedicated; Group 3: challenged and supplemented with dicalzuril, 1 ppm; and Group 4: challenged and supplemented with ZnO-NPs, 20 ppm.

Total protein, albumin, and globulin levels in all challenged groups did not significantly differ from those of the control (Group 1) on the sixth day post-challenge. However, on the fourth week post-challenge, Group 2 had significantly lower total protein and globulin levels than Group 1, while Group 3 and 4 showed a significant increase in their levels (*p* ≤ 0.05) than Group 2 to be comparable to Group 1 (*p* ≥ 0.05) (Figure 8).

#### 3.5.3. Serum Lipid Profile

Serum cholesterol and LDL levels in Group 2 were high (*p* ≤ 0.05), while those of the medicated groups were comparable to Group 1 (*p* ≥ 0.05). There was no effect on the levels of serum triglycerides, HDL, and VLDL among all groups (Table 7).

## 4. Discussion

Zinc (Zn) is a mineral that plays a variety of roles in mammals and birds, including nutrient metabolism, immune system modulation, appetite control, free radical scavenging, and transcription factors in addition to it engaged in the synthesis and/or breakdown of carbohydrates, lipids, proteins, and nucleic acids as a component of numerous enzyme classes [40]. The suggested Zn requirement for broiler chickens is 40mg/kg of food, according to the National Research Council [41]. However, the NRC’s suggested values for most trace minerals are based on earlier broiler strains and may be out of date for today’s commercial broiler strains [42], so to maximize performance, inorganic trace minerals, oxides or sulphates, are traditionally supplied in broiler diets above the NRC approved amount [43]. Therefore, the mineral premix used for the basal diet formulation in this study provided inorganic zinc oxide by 75 mg/kg diet as a nutritional requirement for broiler chickens. Here, we provided additional 20 ppm of ZnO-NPs to infected chicken group with mixed Eimeria species to assess its preventive measure against coccidiosis in comparison to diclazuril. The results of Group 2 revealed that experimental coccidiosis was successfully induced by using 15 times the usual vaccine dose/bird. It resulted in a mortality rate of 11.9% and a significant drop in plasma carotenoid levels, the latter of which may be employed as a marker of coccidial infection [44]. The decreased level of carotenoids could be due to malabsorption resulting from intestinal damage induced by coccidiosis [4]. Besides, alterations in liver function, as shown afterwards here, may help in reducing hepatic storage and release [45]. Furthermore, reactive oxygen species (ROS) generated by coccidiosis may destroy them [46]. On the other hand, carotenoids have antioxidant properties [47]. Therefore, coccidiosis would increase oxidative stress by liberating ROS and impairing carotenoid absorption.

The obtained high MDA and low AOA levels on the fourth week post-challenge were indicators of oxidative stress, which plays a role in coccidiosis pathogenesis [7,48]. The antioxidant efficacy of ZnO-NPs was denoted by the significant increase in the AOA level, which was even more than that of Group 1 (non-infected, basal diet with inorganic zinc oxide), and both medicated groups inhibited oxidative stress on the fourth week post-challenge. Since zinc is a component in free radical scavengers, ZnO-NPs have been proposed to defend against oxidative damage [24,49] induced by coccidiosis.

In Group 2, severe coccidiosis was evidenced by a high oocyst count and lesion score. Although these lesions lasted for 4 weeks post-challenge, the manifestations and lesions were mild indicating that immunity had developed. The medicated groups showed reduced mortality (5.8%) and fecal oocyst number. In addition, the lesion score of Group 3 was statistically decreased in the duodenum but not in the jejunum and ilium. ZnO-NPs remarkably decreased the lesion score in the Jejunum and Ilium, similarly to the effect of diclazuril in the duodenum. Diclazuril, on the other hand, was more effective in preventing caecal lesions than ZnO-NPs. Since diclazuril affects different stages of Eimeria species [50] inhabiting different areas of intestine with variable susceptibility to it, the lesion score along the gut varied in Group 3. As an example, 1 ppm prevented infection with *E. acervulina* and *E. tenella*, whereas up to 2 ppm of diclazuril only reduced *E. maxima*-induced lesion scores [51]. Furthermore, What about the efficacy of diclazuril against *E. mivati*, which is found in the upper section of the small intestine and was included in this challenge? It may need further research. Diclazuril efficacy, on the other hand, was observed not only by the lesion scores but also by improvement in growth and reduction in oocyst shedding. Either medications, diclazuril or ZnO-NPs, inhibited Eimeria replication, as evidenced by a decreased lesion score as well as reduced oocyst shedding. Amer et al. [52] and Abou El-Azm et al. [53] observed similar findings for diclazuril. Dkhil et al. [22] found a significant decrease in the number of *E. papillate* oocysts in the feces of mice treated with ZnO-NPs. Furthermore, its supplementation to *E. steidae*-infected rabbits revealed a non-detectable protozoal stage in the liver [21].

The significantly reduced growth of Group 2 may be due to the nutrient malabsorption, which may have resulted from the reduction in intestinal villi length/width, and crypt depth, and subsequent deviation in various biochemical and enzymatic components [12]. The medicated groups showed a considerable improvement in growth, owing to reduced intestinal lesion scores indicating to improved gut health. These results of diclazuril treatment were corroborate with the results of [53,54]. Furthermore, Zhao et al. [24] and El-Katcha et al. [40] observed that ZnO-NPs were superior to traditional zinc oxide in enhancing feed utilization and growth because zinc is an integral part of more than 200 enzyme systems involved in metabolism [55]. According to Ibrahim et al. [56], ZnO-NPs improved metabolism by enhancing activities of insulin-like growth factors and growth hormone genes. Furthermore, ZnO-NPs improved mucosal villi length/width and crypt depth [40,57], resulting in an increase in intestinal absorptive capacity. As a result, ZnO-NPs may have additional growth-promoting effects.

The PCV%, Hb%, and RBCs count of Group 2 were dramatically decreased, which could have been a result of second generation schizonts rupturing, resulting in substantial damage to the mucosal blood vessels and blood loss [58]. In their study, Ellakany et al. [5] came to similar conclusions. Meanwhile, these indices were significantly increased in Groups 3 and 4. We hypothesize that ZnO-NPs inhibited coccidial proliferation such as diclazuril, thus stopping bleeding, as also reported by El-Shazly et al. [59], who used 2.5 ppm of diclazuril against *E. tenella*, whereas 20 or 40 ppm of ZnO-NPs showed no effect on the hematology of ascites-induced birds [60].

Aside from these changes, the total WBC count did not substantially differ post-challenge. This could be attributed the marked rise in basophils and heterophils and a significant decrease in lymphocytes, resulting in an unchanged WBC count. Infected chickens exhibited a higher heterophil-to-lymphocyte ratio (0.57) than that Group 1 (0.41), indicating stress following the parasitic or viral infections, as observed by Akhtar et al. [61] and El-Shall et al. [62]. There was no change in the percentage of eosinophils and monocytes (*p* ≥ 0.05), which is consistent with the findings of Ellakany et al. [5] and Akhtar et al. [61].

According to Popova-Ralcheva et al. [63], another valid stress indicator in birds is serum cholesterol and LDL levels, which were significantly increased post-challenge (Group 2). However, the medicated groups showed no effect on their levels compared to Group 2. The effects of lipid profile in broiler chickens were previously studied, but without pathogenic challenge [64,65]. Further research is needed to fully comprehend the involvement of cholesterol and other lipids in the pathogenesis of coccidiosis or vice versa.

Kaneko et al. [66] suggested the role of stress-associated cortisone in protein catabolism, which may explain the significant decrease in the total protein and globulin levels in Group 2 on the fourth week post-challenge. Another cause may be the reduced feed intake or hemorrhage induced by coccidiosis. Mondal et al. [6] reported hypo-proteinemia on the fifth and seventh day post-challenge with *E. tenella* infection, which returned to normal levels on the ninth day post-challenge. Our results of hypo-proteinemia were correlated with impaired growth, continuous oocyst shedding until the 25th day post-challenge, and oxidative stress observed on the fourth week post-challenge.

The chickens in this study were infected with four different Eimeria species, each with its unique pathogenicity, life cycle duration, and immunogenicity. Moreover, the experiment was conducted in floor pens, allowing for reinfection and increased pathogenicity before immunity developed. These factors could explain the long-term negative impact of coccidiosis on blood protein level and other parameters.

In this study, the ALP level was considerably high post-challenge as previously reported by Shekhar et al. [67] and El-Shazly et al. [59]. ALP is associated with osteoblastic activity [68], and the high levels could be related to coccidiosis-related hemorrhage as compensation for blood loss [69]. On the other hand, AST and ALT levels were not significantly different (*p* ≥ 0.05) either in medicated or non-medicated infected groups. However, Mondal et al. [6] found a significant increase in the AST level after *E. tenella* exposure, but the ALT level was unchanged. As a result, the intensity of inflammation, intestinal wall injury, blood loss, and the SGOT profile could be responsible for the contradictory results [70].

*Eimeria* spp. and/or strain pathogenicity, dose, vulnerable age, and breed can influence the outcomes of coccidial infections. Both medications showed no effects on the AST and ALT levels, but they lowered the ALP level and increased the total protein and globulin levels. Diclazuril’s ability to inhibit Eimeria multiplication in the gut could explain the improvement in the evaluated parameters. ZnO-NPs showed antioxidant, growth-promoting, and anticoccidial activities. From another perspective, ZnO-NPs can boost immune responses and infection resistance [71]. The precise mechanism of its anticoccidial action, however, remains unknown. Here, we provided ZnO-NPs as an additional dose over the natural requirement of broiler chickens; so, what about its residue in tissue or its excretion into the environment? Furthermore, if we totally resubstitute inorganic zinc with ZnO-NPs in basal diet for chickens, will they show an anticoccidial effect? All of these questions require further research.

## 5. Conclusions

The parasite and growth indicators used to determine the severity of infection of mixed Eimeria species in chickens were both positive for coccidial infectivity. ZnO-NP supplementation of 20 ppm in the basal diet originally contains 75 mg/kg of inorganic zinc oxide, improved growth performance, intestinal lesions, plasma carotenoids, and hematological indices as well as demonstrated substantial antioxidant activity and enhanced survival rate in infected chickens. Interestingly, the fecal oocyst shedding also decreased. The anticoccidial activity of 20 ppm of ZnO-NPs could be comparable to that of 1 ppm of diclazuril.

## Figures and Tables

**Table 1:** The composition % and chemical analysis of basal diet.

Ingredients	Starter Ration (1st–21st Day of Age)	Grower Ration (22nd–42nd Day of Age)
Yellow corn %	56	60.5
Soybean meal %	35	29.1
Corn gluten meal %	5	5
Limestone %	1.5	1.5
dicalcium phosphate %	1	0.8
Carbonate %	0.075	0.12
NaCl %	0.3	0.3
Vitamins premix ^1^ %	0.15	0.15
Trace mineral premix ^2^ %	0.15	0.15
DL-Methionine %	0.15	0.15
L-Lysine %	0.25	0.25
Vegetable oil %	0.5	1.5
Total	100	100
Chemical analysis %		
Crude protein	23	21.16
ME Energy Kcal/Kg ration	2931.3	3042.08
Calcium	0.92	0.88
Available phosphorous	0.46	0.44
Lysine	1.45	1.29
Methionine	0.56	0.53
Ash	5.98	5.51

^1^ Produced by Nutristar international Co. and provided per 1 kg diet: vitamin A: 18,000.0 IU, vitamin D3: 3750.0 IU, vitamin E: 15.0 mg, vitamin K3: 1.5 mg, vitamin B1: 1.5 mg, vitamin B2: 7.5 mg, vitamin B6: 2.25 mg, vitamin B12: 15.0 µg, niacin: 45.0 mg, biotin: 75.0 µg, folic acid: 1.5 mg, pantothenic acid: 15.0 mg, antioxidant-ethoxyquin: 375.0 µg. ^2^ Produced by Nutristar international Co. and and provided per 1 kg diet: Mn (Mn oxide): 90.0 mg, Zn (Zn oxide): 75.0 mg, Cu (copper sulphate): 7.5 mg, Fe (Fe chloride): 45.0 mg, I (calcium iodate): 750.0 µg, Se (sodium selenate): 150 µg and Co (cobalt sulphate): 150 µg.

**Table 2:** Relative organ to body weight index post-coccidial challenge.

Chicken Groups	Organ Index					
	Liver	Kidney	Gizzard	Heart	Spleen	Bursa of Fabricius
6th day post challenge						
Group1	2.75 ± 0.04	0.54 ± 0.06	2.46 ± 0.16	0.71 ± 0.01	0.19 ± 0.00	0.20 ± 0.01
Group 2	3.14 ± 0.08	0.56 ± 0.04	2.58 ± 0.09	0.70 ± 0.03	0.25 ± 0.02	0.14 ± 0.01
Group 3	2.90 ± 0.22	0.58 ± 0.05	2.62 ± 0.16	0.63 ± 0.03	0.18 ± 0.02	0.18 ± 0.01
Group 4	3.10 ± 0.04	0.48 ± 0.03	2.75 ± 0.08	0.66 ± 0.03	0.18 ± 0.01	0.15 ± 0.01
4th week post challenge						
Group1	2.88 ± 0.08	0.52 ± 0.01	1.36 ± 0.02	0.46 ± 0.15	0.17 ± 0.01	0.08 ± 0.01 ^ab^
Group 2	2.45 ± 0.09	0.50 ± 0.03	1.40 ± 0.03	0.50 ± 0.03	0.13 ± 0.01	0.07 ± 0.00 ^b^
Group 3	2.48 ± 0.02	0.50 ± 0.04	1.32 ± 0.06	0.43 ± 0.01	0.14 ± 0.04	0.10 ± 0.01 ^ab^
Group 4	2.29 ± 0.04	0.47 ± 0.01	1.47 ± 0.06	0.53 ± 0.00	0.14 ± 0.01	0.12 ± 0.02 ^a^

Group 1: unchallenged and unmedicated; Group 2: challenged and unmedicated; Group 3: challenged and supplemented with dicalzuril, 1ppm; and Group 4: challenged and supplemented with ZnO-NPs, 20 ppm. Means within a column with different superscripts differ (*p* ≤ 0.05) at time part.

**Table 3:** Growth performance at weekly intervals post-coccidal challenge and mortality %.

Experimental Groups	Parameter	Age of Birds (Days (d)) *					Mortality % (No. of Dead Birds/Total **)
		14th–21st d	21st–28th d	28th–35th d	35th–42nd d	(First–42nd d)	
Group 1	B.wt gain (g)	390.33 ± 8.38 ^x^	497.55 ± 8.1 ^x^	555.76 ± 12.25 ^xy^	581.82 ± 16.98 ^x^	1995.05 ± 121.68 ^x^	0% (0)
	FCR (g/g)	1.37 ± 0.03 ^b^	1.53 ± 0.02 ^c^	1.87 ± 0.04 ^b^	2.19 ± 0.03 ^ab^	1.68 ± 0.03 ^b^	
Group 2	B.wt gain (g)	177.86 ± 11.86 ^z^	328.94 ± 16.4 ^z^	525.97 ± 22.016 ^y^	401.45 ± 14.32 ^y^	1466.29 ± 108.31 ^y^	11.9% (4/34)
	FCR (g/g)	2.78 ± 0.25 ^a^	2.43 ± 0.14 ^a^	2.25 ± 0.12 ^a^	2.15 ± 0.10 ^ab^	2.15 ± 0.10 ^a^	
Group 3	B.wt gain (g)	283.33 ± 8.30 ^y^	345.71 ± 9.28 ^z^	617.94 ± 13.93 ^x^	544.24 ± 16.74 ^x^	1806.88 ± 110.70 ^x^	5.8% (2/34)
	FCR (g/g)	1.60 ± 0.07 ^b^	1.98 ± 0.06 ^b^	1.60 ± 0.03 ^b^	2.0 ± 0.06 ^b^	1.71 ± 0.04 ^b^	
Group 4	B.wt gain (g)	252.30 ± 9.32 ^y^	452.94 ± 9.44 ^y^	610.94 ± 15.62 ^x^	539.17 ± 13.60 ^x^	1757.88 ± 125.94 ^x^	5.8% (2/34)
	FCR (g/g)	1.71 ± 0.06 ^b^	1.69 ± 0.04 ^bc^	1.67 ± 0.05 ^b^	2.30 ± 0.06 ^a^	1.73 ± 0.03 ^b^	

Group 1: unchallenged and unmedicated; Group 2: challenged and unmedicated; Group 3: challenged and supplemented with dicalzuril, 1 ppm; and Group 4: challenged and supplemented with ZnO-NPs, 20 ppm. * Coccidial challenge was conducted on the 14th day of age. Means within a column with different superscripts differ (*p* < 0.05) for each parameter (abc for FCR; xyz for B.wt gain). ** Death of birds occurred after 6th d post-coccidial challenge at which 6 birds were killed for evaluating parameters, so the mortality % was calculated from 34 birds/group.

**Table 4:** Hematological parameters of chicken groups post-coccidial challenge (Mean ± SE).

Experimental Groups	6th Day Post Challenge			4th Week Post Challenge		
	PCV (%)	RBCs (10^6^/mm^3^)	Hb (mg/dL)	PCV%	RBCs (10^6^/mm^3^)	Hb (mg/dL)
Group 1	30.30 ± 0.19 ^ab^	3.48 ± 0.02 ^ab^	10.12 ± 0.07 ^ab^	38.20 ± 1.12 ^a^	4.36 ± 0.12 ^a^	12.65 ± 0.37 ^a^
Group 2	21.89 ± 0.43 ^c^	2.42 ± 0.09 ^c^	7.24 ± 0.14 ^c^	30.90 ± 1.85 ^b^	3.10 ± 0.31 ^b^	10.30 ± 0.62 ^b^
Group 3	27.44 ± 0.52 ^b^	3.22 ± 0.08 ^b^	9.11 ± 0.18 ^b^	37.44 ± 1.36 ^a^	4.18 ± 0.10 ^a^	12.58 ± 0.52 ^a^
Group 4	32.22 ± 2.33 ^a^	3.74 ± 0.29 ^a^	10.84 ± 0.84 ^a^	36.18 ± 1.56 ^a^	3.96 ± 0.23 ^a^	11.35 ± 0.77 ^ab^

Group 1: unchallenged and unmedicated; Group 2: challenged and unmedicated; Group 3: challenged and supplemented with dicalzuril, 1 ppm; and Group 4: challenged and supplemented with ZnO-NPs, 20 ppm. Means within a column with different superscripts differ (*p* ≤ 0.05).

**Table 5:** Differential leukocytes count in chicken groups post-coccidial challenge (Mean ± SE).

Experimental Groups	Leukocytes (×10^3^/cmm)	Basophils %	Eosinophils %	Heterophils%	Lymphocytes%	Monocytes%
6th day post challenge						
Group1	5.07 ± 0.13	0.00 ± 0.00 ^b^	1.00 ± 0.00	29.00 ± 0.05 ^c^	65.80 ± 0.20 ^a^	4.20 ± 0.37
Group 2	5.11 ± 0.78	0.60 ± 0.24 ^a^	1.00 ± 0.00	34.60 ± 1.86 ^a^	59.80 ± 2.63 ^b^	4.00 ± 0.63
Group 3	5.66 ± 0.44	0.20 ± 0.20 ^ab^	1.00 ± 0.00	30.60 ± 1.36 ^b^	63.40 ± 1.47 ^ab^	4.80 ± 0.58
Group 4	4.66 ± 0.40	0.00 ± 0.00 ^b^	1.00 ± 0.00	34.20 ± 1.91 ^a^	60.80 ± 1.43 ^ab^	4.00 ± 0.71
4th week post challenge						
Group1	5.80 ± 0.52	0.00 ± 0.00	1.00 ± 0.00	33.80 ± 1.46	59.60 ± 1.72	5.60 ± 0.40
Group 2	7.16 ± 0.74	0.00 ± 0.00	1.00 ± 0.00	36.20 ± 0.80	57.80 ± 0.66	5.00 ± 0.32
Group 3	7.24 ± 0.85	0.00 ± 0.00	1.00 ± 0.00	32.00 ± 1.30	61.80 ± 1.71	5.20 ± 0.49
Group 4	5.32 ± 0.98	0.00 ± 0.00	1.00 ± 0.00	32.40 ± 2.25	60.80 ± 2.06	5.80 ± 0.73

Group 1: unchallenged and unmedicated; Group 2: challenged and unmedicated; Group 3: challenged and supplemented with dicalzuril, 1 ppm; and Group 4: challenged and supplemented with ZnO-NPs, 20 ppm. Means within a column with different superscripts differ (*p* ≤ 0.05).

**Table 6:** Liver enzymes post-coccidial challenge in chicken groups (Mean ± SE).

Experimental Groups	Liver Function			
	AST U/L	ALT (U/L)	ALP (U/l)	Bilirubin
6th day post challenge				
Group1	69.48 ± 2.57	31.10 ± 1.11	37.12 ± 4.33 ^b^	1.12 ± 0.02
Group 2	67.68 ± 1.04	32.80 ± 1.45	71.62 ± 22.64 ^a^	0.81 ± 0.11
Group 3	71.64 ± 2.75	33.60 ± 1.49	43.86 ± 4.39 ^b^	0.96 ± 0.08
Group 4	72.67 ± 0.77	34.20 ± 1.44	44.18 ± 15.12 ^b^	0.97 ± 0.17
4th week post challenge				
Group1	69.02 ± 1.33	35.80 ± 1.44	43.79 ± 4.65 ^b^	0.89 ± 0.01
Group 2	69.24 ± 1.32	36.80 ± 2.37	89.10 ± 4.85 ^a^	0.73 ± 0.04
Group 3	66.72 ± 1.2	34.10 ± 1.96	67.31 ± 13.38 ^b^	0.80 ± 0.04
Group 4	69.66 ± 1.23	31.50 ± 1.01	46.83 ± 6.29 ^b^	0.82 ± 0.02

Group 1: unchallenged and unmedicated; Group 2: challenged and unmedicated; Group 3: challenged and supplemented with dicalzuril, 1 ppm; and Group 4: challenged and supplemented with ZnO-NPs, 20 ppm. AST: Aspartate Aminotransferase, ALT: Alanine Aminotransferase, ALP: Serum Alkaline Phosphates. Means within a column with different superscripts differ (*p* ≤ 0.05).

**Table 7:** Serum lipid profile of the experimental groups post-challenge with mixed Eimeria species (Mean ± SE).

Experimental Groups	Serum Level (mg/dL)				
	Total Cholesterol	Triglyceride	HDL	LDL	VLDL
6th day post challenge					
Group1	201.25 ± 3.90 ^b^	201.09 ± 1.07	49.70 ± 1.27	111.17 ± 3.18 ^b^	40.38 ± 0.27
Group 2	219.99 ± 1.35 ^a^	199.72 ± 1.17	48.46 ± 1.16	131.59 ± 2.31 ^a^	39.94 ± 0.23
Group 3	218.34 ± 2.00 ^a^	199.75 ± 1.32	47.30 ± 1.17	131.09 ± 2.86 ^a^	39.95 ± 0.27
Group 4	218.00 ± 1.40 ^a^	201.12 ± 1.15	48.18 ± 1.57	130.59 ± 1.61 ^a^	40.23 ± 0.23
4th week post challenge					
Group1	207.17 ± 0.66 ^b^	197.55 ± 0.70	50.84 ± 0.30	116.82 ± 0.50 ^b^	39.51 ± 0.14
Group 2	216.47 ± 0.94 ^a^	200.51 ± 1.14	49.94 ± 1.33	126.43 ± 2.19 ^a^	40.10 ± 0.23
Group 3	214.27 ± 2.49 ^a^	198.27 ± 0.96	50.26 ± 2.21	124.36 ± 5.75 ^a^	39.65 ± 0.19
Group 4	207.16 ± 0.91 ^b^	198.92 ± 0.71	50.48 ± 1.99	126.90 ± 2.21 ^a^	39.78 ± 0.14

Group 1: unchallenged and unmedicated; Group 2: challenged and unmedicated; Group 3: challenged and supplemented with dicalzuril, 1 ppm; and Group 4: challenged and supplemented with ZnO-NPs, 20 ppm. Means within a column with different superscripts differ (*p* ≤ 0.05).
